# Patterns of comorbidities in patients with atrial fibrillation and impact on management and long-term prognosis: an analysis from the Prospective Global GLORIA-AF Registry

**DOI:** 10.1186/s12916-024-03373-4

**Published:** 2024-04-08

**Authors:** Giulio Francesco Romiti, Bernadette Corica, Davide Antonio Mei, Arnaud Bisson, Giuseppe Boriani, Brian Olshansky, Tze-Fan Chao, Menno V. Huisman, Marco Proietti, Gregory Y. H. Lip, on behalf of the GLORIA-AF Investigators

**Affiliations:** 1 Liverpool Centre for Cardiovascular Science, Institute of Ageing and Chronic Disease, University of Liverpool, William Henry Duncan Building, 6 West Derby Street, Liverpool, L7 8TX UK; 2 Department of Translational and Precision Medicine, Sapienza – University of Rome, Rome, Italy; 3 Cardiology Division, Department of Biomedical, Metabolic and Neural Sciences, University of Modena and Reggio Emilia, Policlinico di Modena, Modena, Italy; 4 Service de Cardiologie, Centre Hospitalier Régional Universitaire et Faculté de Médecine de Tours, Tours, France; 5 Division of Cardiology, Department of Medicine, University of Iowa, Iowa City, USA; 6 Division of Cardiology, Department of Medicine, Taipei Veterans General Hospital, Taipei, Taiwan; 7 Institute of Clinical Medicine, and Cardiovascular Research Center, National Yang Ming Chiao Tung University, Taipei, Taiwan; 8 Department of Thrombosis and Hemostasis, Leiden University Medical Center, Leiden, the Netherlands; 9 Department of Clinical Sciences and Community Health, University of Milan, Milan, Italy; 10 Division of Subacute Care, IRCCS Istituti Clinici Scientifici Maugeri, Milan, Italy; 11 Danish Center for Health Services Research, Department of Clinical Medicine, Aalborg University, Aalborg, Denmark

**Keywords:** Atrial fibrillation, Comorbidities, Multimorbidity, Clinical complexity

## Abstract

**Background:**

Clinical complexity, as the interaction between ageing, frailty, multimorbidity and polypharmacy, is an increasing concern in patients with AF. There remains uncertainty regarding how combinations of comorbidities influence management and prognosis of patients with atrial fibrillation (AF). We aimed to identify phenotypes of AF patients according to comorbidities and to assess associations between comorbidity patterns, drug use and risk of major outcomes.

**Methods:**

From the prospective GLORIA-AF Registry, we performed a latent class analysis based on 18 diseases, encompassing cardiovascular, metabolic, respiratory and other conditions; we then analysed the association between phenotypes of patients and (i) treatments received and (ii) the risk of major outcomes. Primary outcome was the composite of all-cause death and major adverse cardiovascular events (MACE). Secondary exploratory outcomes were also analysed.

**Results:**

32,560 AF patients (mean age 70.0 ± 10.5 years, 45.4% females) were included. We identified 6 phenotypes: (i) low complexity (39.2% of patients); (ii) cardiovascular (CV) risk factors (28.2%); (iii) atherosclerotic (10.2%); (iv) thromboembolic (8.1%); (v) cardiometabolic (7.6%) and (vi) high complexity (6.6%). Higher use of oral anticoagulants was found in more complex groups, with highest magnitude observed for the cardiometabolic and high complexity phenotypes (odds ratio and 95% confidence interval CI): 1.76 [1.49–2.09] and 1.57 [1.35–1.81], respectively); similar results were observed for beta-blockers and verapamil or diltiazem. We found higher risk of the primary outcome in all phenotypes, except the CV risk factor one, with highest risk observed for the cardiometabolic and high complexity groups (hazard ratio and 95%CI: 1.37 [1.13–1.67] and 1.47 [1.24–1.75], respectively).

**Conclusions:**

Comorbidities influence management and long-term prognosis of patients with AF. Patients with complex phenotypes may require comprehensive and holistic approaches to improve their prognosis.

**Supplementary Information:**

The online version contains supplementary material available at 10.1186/s12916-024-03373-4.

## Background

Atrial fibrillation (AF) frequently occurs in older patients with multiple comorbidities. Indeed, multimorbidity (defined as the presence of two or more concurrent diseases [1]) is common in patients with AF: most patients currently show four or more conditions when AF is diagnosed—a steep increase compared to 20 years ago [2]. Multimorbidity has a significant impact on the natural history of AF, with detrimental effects on prognosis, as well as influence on healthcare-associated costs and the quality of overall management (including stroke prevention) [3–6]. For these reasons, evaluating and addressing multimorbidity has become central in the clinical management of AF [2, 3, 7], also in view of the association with other clinical risk factors. Indeed, multimorbidity—along with ageing, frailty and polypharmacy—contributes to the so-called clinical complexity state [8, 9], a scenario in which the detrimental interplay between different determinants (e.g. complex comorbidities patterns, interaction of several drugs, older age and frailty) concur to influence prognosis and bolster the risk of adverse outcomes. In patients with AF, clinical complexity has been previously linked with suboptimal evidence-based management and worse outcomes [6, 10], underlying its effect on the natural history of AF.

Given the central role of multimorbidity in determining clinical complexity, the understanding of its epidemiology is crucial in patients with AF. Nonetheless, the current definition of multimorbidity does not capture the complexity arising from the different *combinations* of comorbidities: chronic long-term conditions (both cardiovascular and non-cardiovascular) tend to occur together in clusters, often with heterogenous patterns and unpredictable—yet usually synergistic—detrimental effects on prognosis [6, 8–10]. To date, however, there is no clear understanding of how comorbidities aggregate in patients with AF, and how these interactions influence management and prognosis [1, 2]. Latent class analysis (LCA) is an unsupervised clustering and model-based approach that identifies subgroups of individuals (i.e. the *latent classes*) who have similar characteristics, based on a set of variables [11, 12]. This approach has been previously used to identify clinical phenotypes and multimorbidity patterns in various populations [13–15].

In this ancillary analysis from *Global Registry on Long-Term Antithrombotic Treatment in Patients with Atrial Fibrillation* (GLORIA-AF) Phase II and Phase III Registry, we performed a LCA to explore phenotypes of AF patients according to comorbidity patterns and analysed the association between such phenotypes and management and prognosis of AF.

## Methods

The GLORIA-AF is a prospective, multicentre and international registry programme structured in three phases, aimed to assess the long-term real-world safety and efficacy of dabigatran etexilate in patients with AF. Details on the design, follow-up and primary results of GLORIA-AF registry have been previously reported [16–19]. Briefly, during the study period (2011–2014 for phase II and 2014–2016 for phase III), patients (≥18 years old) with a recent diagnosis of non-valvular AF (i.e. within 3 months or 4.5 months in Latin America) and a CHA_2_DS_2_-VASc score ≥1 were consecutively enrolled. All patients provided written informed consent. The main exclusion criteria were AF due to a reversible cause, mechanical heart valve (or patients expected to undergo valve replacement), having received vitamin K antagonist (VKA) for >60 days during lifetime or other clinical indication for oral anticoagulant (OAC) and short life expectancy (<1 year). The study protocol was approved by local institutional review boards at each participating centre. The study was conducted according to the Good Clinical Practice and the Declaration of Helsinki. The original studies were registered with ClinicalTrials.gov, NCT01468701, NCT01671007 and NCT01937377.

At baseline, investigators recorded data regarding demographics, comorbidities and treatment prescribed for all patients recruited, using standardised case report forms (CRF).

### Comorbidities and treatments

For this exploratory analysis, we considered 18 diseases and conditions, among those recorded at baseline. Cardiovascular conditions included arterial hypertension, coronary artery disease (CAD), heart failure (HF), peripheral artery disease (PAD), history of previous stroke/transient ischemic attack (TIA), history of venous thromboembolism and previous bleeding events. We also included non-cardiovascular conditions, i.e. diabetes mellitus, hyperlipidemia, obesity, history of cancer, abnormal kidney function (defined as chronic dialysis, renal transplantation, or serum creatinine ≥200 μmol/L), chronic obstructive pulmonary disease (COPD), emphysema, hyperthyroidism, liver disease, gastrointestinal disease (including peptic disease, heartburn/pyrosis and other abdominal conditions) and the presence of neurologic conditions (as recorded by investigator in the CRF). Investigators were able to record whether patients included had one of more of each condition, along with information regarding treatment prescribed. For this analysis, we considered use of antithrombotics, as well as concomitant treatment with cardiovascular drugs (i.e. angiotensin converting enzyme (ACE) inhibitors, angiotensin receptor blockers, diuretics, beta-blockers, digoxin, verapamil/diltiazem, class IC antiarrhythmic drugs (which included propafenone and flecainide), amiodarone/dronedarone, other antiarrhythmics) and non-cardiovascular drugs (i.e. oral hypoglycaemic agents, insulin, proton pump inhibitors and statins).

### Follow-up and outcomes

During phase II, a 2-year follow-up was performed only for patients prescribed dabigatran at baseline. During phase III, all patients (regardless of antithrombotic therapy received) were followed-up for 3 years. OAC discontinuation and major clinical outcomes were recorded during follow-up. We analysed treatment discontinuation at 24 months only for those patients who received OAC at baseline. As per previous analyses [20], discontinuation was defined as switching to another antithrombotic regimen (including switching to a different OAC) or interruption ≥30 days of treatment received at baseline. Non-persistence was defined as OAC discontinuation or study termination.

We defined the *primary outcome* as the composite of all-cause death and major adverse cardiovascular events (MACE, which included cardiovascular death, stroke and myocardial infarction). Secondary exploratory outcomes included: (i) all-cause mortality, (ii) cardiovascular mortality, (iii) MACE (as previously defined), (iv) thromboembolism (i.e. the composite of stroke, TIA and other non-central nervous system thromboembolism) and (v) major bleeding (defined as a life-threatening or fatal bleeding, symptomatic bleeding in a critical organ or a bleeding associated with a haemoglobin reduction of ≥20 g/L or leading to ≥2 units of blood transfusion).

### Statistical analysis

A graphical representation of the workflow of this analysis is reported in Additional file 1: Figure S1. We performed an exploratory latent class analysis based on the 18 conditions described above, using the ‘poLCA’ package in R [21]. The optimal number of classes was selected according to the Bayesian Information Criterion (BIC) and the consistent Akaike Information Criterion (cAIC), with lower values indicating better fit [22], and also according to clinical judgement. Posterior probability of membership was calculated for each patient, and for further analyses, each subject was then assigned to one of the latent classes, according to the modal posterior probability of membership. The classes identified were then named, considering the most relevant clinical characteristics, and the prevalence of comorbidities. Baseline characteristics were then computed and reported according to the groups identified.

Continuous variables were reported as mean and standard deviation (SD) or median and interquartile range (IQR); normally distributed variables were compared using parametric test, while non-normally distributed variables were compared using non-parametric tests. Binary and categorical variables were reported as frequencies and percentages, and Chi-square test was used for comparison.

The association between latent classes and drugs prescriptions was evaluated using a multiple logistic regression model, with components of CHA_2_DS_2_-VASc score (age <65, 65–75 or ≥75 years, sex, hypertension, diabetes, HF, CAD, history of stroke/TIA and PAD), phase of recruitment, type of AF (paroxysmal, persistent or permanent), BMI, and history of previous bleeding as covariates. Results were reported as odds ratios (OR) and 95% confidence intervals (CI).

The associations with OAC discontinuation and major outcomes were assessed using Cox-regression models, with the same covariates used in the logistic regression model. Additionally, the regression models for the risk of major outcomes were also adjusted for the use of OAC. Results were reported as hazard ratios (HR) with 95% confidence intervals (CI). For the primary composite outcome, we additionally reported Kaplan–Meier curves, and survival distributions were compared using the log-rank test. A two-sided *p* < 0.05 was considered statistically significant. All the analyses were performed using R 4.3.1 (R Core Team 2020, Vienna, Austria).

## Results

32,560 patients enrolled in the GLORIA-AF phase II and phase III (mean age 70.0 ± 10.5 years, 45.4% females) and who had available data on the 18 conditions and diseases used in the LCA were included in this analysis.

### Phenotypes of patients based on comorbidity patterns

**Fig. 1:**
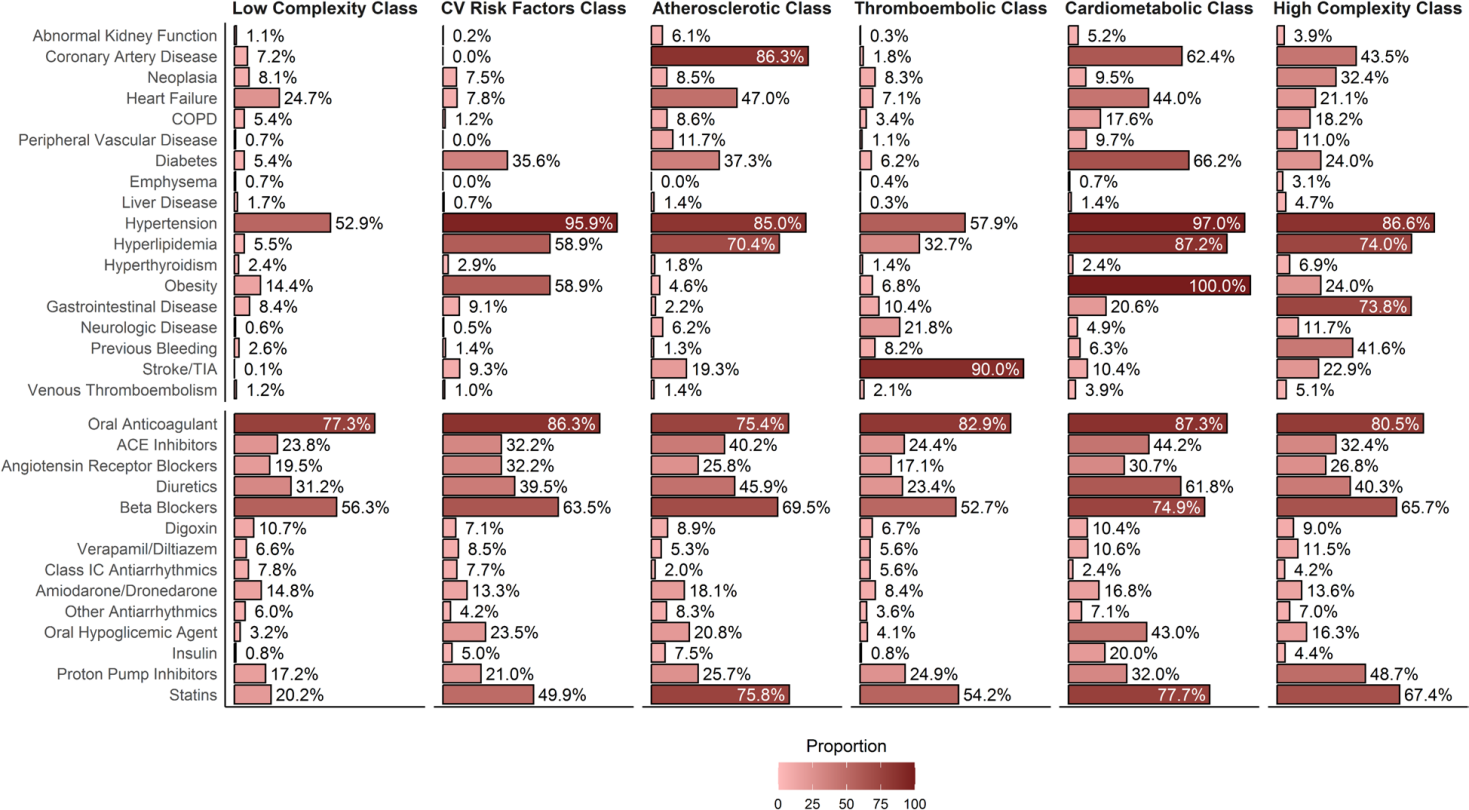
Prevalence of comorbidities and drugs prescribed at baseline according to latent classes. ACE angiotensin converting enzyme; COPD chronic obstructive pulmonary disease, TIA transient ischemic attack

Baseline characteristics according to latent class allocation are reported in Table 1; a synoptic view of comorbidities’ prevalence, and proportion of patients prescribed with each drug class is shown in Fig. 1.

**Table 1:** Baseline characteristics according to latent classes

Variables, ***n*** (%)	Low complexity class (***n*** = 12,774)	CV risk factors class (***n*** = 9195)	Atherosclerotic class (***n*** = 3328)	Thromboembolic class (***n*** = 2647)	Cardiometabolic class (***n*** = 2462)	High complexity phenotype (***n*** = 2154)	***p***
Age, mean (SD)	69.2 (11.1)	68.8 (10.4)	73.1 (9.3)	72.8 (10.2)	69.4 (9.3)	73.3 (8.9)	<0.001
<65 years	3640/12,774 (28.5)	2859/9195 (31.1)	568/3328 (17.1)	469/2647 (17.7)	716/2462 (29.1)	330/2154 (15.3)	
≥75 years	4634/12,774 (36.3)	2993/9195 (32.6)	1661/3328 (49.9)	1349/2647 (51.0)	782/2462 (31.8)	1064/2154 (49.4)	
65 to <75 years	4500/12,774 (35.2)	3343/9195 (36.4)	1099/3328 (33.0)	829/2647 (31.3)	964/2462 (39.2)	760/2154 (35.3)	
Females	6047/12,774 (47.3)	4560/9195 (49.6)	1128/3328 (33.9)	1188/2647 (44.9)	934/2462 (37.9)	940/2154 (43.6)	<0.001
Region of recruitment							<0.001
North America	1943/12,774 (15.2)	2750/9195 (29.9)	842/3328 (25.3)	355/2647 (13.4)	1240/2462 (50.4)	901/2154 (41.8)	
Europe	6099/12,774 (47.7)	4350/9195 (47.3)	1395/3328 (41.9)	1582/2647 (59.8)	874/2462 (35.5)	800/2154 (37.1)	
Latin America	1040/12,774 (8.1)	673/9195 (7.3)	178/3328 (5.3)	161/2647 (6.1)	120/2462 (4.9)	72/2154 (3.3)	
Africa/Middle East	128/12,774 (1.0)	163/9195 (1.8)	83/3328 (2.5)	22/2647 (0.8)	69/2462 (2.8)	22/2154 (1.0)	
Asia	3564/12,774 (27.9)	1259/9195 (13.7)	830/3328 (24.9)	527/2647 (19.9)	159/2462 (6.5)	359/2154 (16.7)	
Recruited in phase III	7551/12,774 (59.1)	5476/9195 (59.6)	1921/3328 (57.7)	1594/2647 (60.2)	1384/2462 (56.2)	1249/2154 (58.0)	0.017
BMI, kg/m^2^, median [IQR]	25.8 [23.3–28.4]	30.9 [27.0–34.6]	26.0 [24.0–28.1]	25.5 [23.2–27.8]	34.3 [31.7–38.2]	27.1 [24.2–29.8]	<0.001
Paroxysmal AF	6800/12,774 (53.2)	5184/9195 (56.4)	1919/3328 (57.7)	1538/2647 (58.1)	1355/2462 (55.0)	1326/2154 (61.6)	<0.001
Comorbidities							
Number of comorbidities, median [IQR]	1 [1–2]	3 [2–4]	4 [3–5]	3 [2–3]	5 [5–6]	5 [4–6]	<0.001
Hypertension	6759/12,774 (52.9)	8819/9195 (95.9)	2829/3328 (85.0)	1533/2647 (57.9)	2389/2462 (97.0)	1865/2154 (86.6)	<0.001
CHF	3159/12,774 (24.7)	718/9195 (7.8)	1563/3328 (47.0)	188/2647 (7.1)	1084/2462 (44.0)	455/2154 (21.1)	<0.001
CAD	920/12,774 (7.2)	0/9195 (0.0)	2873/3328 (86.3)	48/2647 (1.8)	1537/2462 (62.4)	936/2154 (43.5)	<0.001
Diabetes mellitus	692/12,774 (5.4)	3272/9195 (35.6)	1243/3328 (37.3)	164/2647 (6.2)	1629/2462 (66.2)	517/2154 (24.0)	<0.001
PAD	90/12,774 (0.7)	0/9195 (0.0)	391/3328 (11.7)	28/2647 (1.1)	239/2462 (9.7)	237/2154 (11.0)	<0.001
History of stroke/TIA	12/12,774 (0.1)	859/9195 (9.3)	642/3328 (19.3)	2382/2647 (90.0)	255/2462 (10.4)	494/2154 (22.9)	<0.001
Previous bleeding	333/12,774 (2.6)	129/9195 (1.4)	42/3328 (1.3)	217/2647 (8.2)	156/2462 (6.3)	897/2154 (41.6)	<0.001
Abnormal kidney function	139/12,774 (1.1)	15/9195 (0.2)	203/3328 (6.1)	9/2647 (0.3)	128/2462 (5.2)	84/2154 (3.9)	<0.001
Neoplasia	1040/12,774 (8.1)	690/9195 (7.5)	284/3328 (8.5)	219/2647 (8.3)	234/2462 (9.5)	697/2154 (32.4)	<0.001
COPD	690/12,774 (5.4)	106/9195 (1.2)	286/3328 (8.6)	90/2647 (3.4)	434/2462 (17.6)	391/2154 (18.2)	<0.001
Emphysema	88/12,774 (0.7)	0/9195 (0.0)	0/3328 (0.0)	10/2647 (0.4)	17/2462 (0.7)	66/2154 (3.1)	<0.001
Hepatic disease	217/12,774 (1.7)	66/9195 (0.7)	47/3328 (1.4)	9/2647 (0.3)	35/2462 (1.4)	102/2154 (4.7)	<0.001
Hyperlipidemia	705/12,774 (5.5)	5419/9195 (58.9)	2342/3328 (70.4)	865/2647 (32.7)	2148/2462 (87.2)	1595/2154 (74.0)	<0.001
Hyperthyroidism	308/12,774 (2.4)	268/9195 (2.9)	59/3328 (1.8)	37/2647 (1.4)	59/2462 (2.4)	149/2154 (6.9)	<0.001
Obesity	1840/12,774 (14.4)	5417/9195 (58.9)	152/3328 (4.6)	181/2647 (6.8)	2462/2462 (100.0)	518/2154 (24.0)	<0.001
Gastrointestinal diseases	1073/12,774 (8.4)	834/9195 (9.1)	73/3328 (2.2)	275/2647 (10.4)	508/2462 (20.6)	1590/2154 (73.8)	<0.001
Neurologic disease	72/12,774 (0.6)	44/9195 (0.5)	207/3328 (6.2)	577/2647 (21.8)	121/2462 (4.9)	253/2154 (11.7)	<0.001
Previous VTE	154/12,774 (1.2)	89/9195 (1.0)	46/3328 (1.4)	56/2647 (2.1)	97/2462 (3.9)	110/2154 (5.1)	<0.001
Risk Scores							
CHA_2_DS_2_-VASc, median [IQR]	2.00 [2.00, 3.00]	3.00 [2.00, 4.00]	4.00 [3.00, 5.00]	4.00 [3.00, 5.00]	4.00 [3.00, 5.00]	4.00 [3.00, 5.00]	<0.001
HAS-BLED, median [IQR]	1.00 [1.00, 2.00]	1.00 [1.00, 2.00]	2.00 [1.00, 2.00]	2.00 [1.00, 3.00]	1.00 [1.00, 2.00]	2.00 [1.00, 3.00]	<0.001

*AF* atrial fibrillation, *BMI* body mass index, *CAD* coronary artery disease, *CHF* congestive heart failure, *COPD* chronic obstructive pulmonary disease, *EHRA* European Heart Rhythm Association, *IQR* interquartile range, *PAD* peripheral artery disease, *SD* standard deviation, *TIA* transient ischemic attack, *VTE* venous thromboembolism. *p* values are for all levels

The largest group was represented by the ‘low complexity’ phenotype (*n* = 12,774, 39.2%), defined by a low prevalence of most comorbid conditions, except for hypertension (52.9%) and HF (24.7%), and the lowest median number of comorbidities (1 [IQR 1–2]). Patients included in the ‘cardiovascular (CV) risk factors’ group (*n* = 9195, 28.2%; median number of comorbidities: 3 [IQR: 2–4]) were the youngest and had high prevalence of hypertension (95.9%), obesity and hyperlipidaemia (58.9% each), with also a considerable prevalence of diabetes mellitus (35.6%). The ‘atherosclerotic’ class (*n* = 3328, 10.2%; median number of comorbidities: 4 [IQR: 3–5]), conversely, had a high prevalence of CAD, HF and hyperlipidaemia and also showed the highest prevalence of PAD (11.7%) and the lowest female representation (33.9%). We also identified a ‘thromboembolic’ class (*n* = 2647, 8.1%, median number of comorbidities: 3 [IQR 2–3]), with 90% of patients with history of previous stroke/TIA, and a ‘cardiometabolic’ class (*n* = 2462, 7.6%, median number of comorbidities 5 [IQR 5–6]), mostly composed of obese subjects with high prevalence of both cardiovascular and metabolic conditions. Finally, the ‘high complexity’ class (*n* = 2154, 6.6%, median number of comorbidities 5 [IQR: 4–6]) had a significant burden of both cardiovascular and non-cardiovascular conditions, including gastrointestinal diseases (73.8%), history of bleeding (41.6%), cancer (32.4%) and COPD (18.2%).

### Pharmacological treatments and OAC discontinuation

Treatments received according to comorbidities phenotypes are reported in Additional file 1: Table S2. Additionally, antithrombotic treatment according to phenotypes are shown in Additional file 1: Figure S2.

OAC were largely used in all groups, with the ‘CV risk factors’ and the cardiometabolic groups showing the highest rates of OAC use (86.3% and 87.3%, respectively); use of OAC was lowest among patients in the atherosclerotic class (75.4%). The highest rate of non-vitamin K antagonist oral anticoagulant (NOAC) use was observed in the CV risk factor class (61.1%).

Regarding other treatments, the ‘CV risk factors’, atherosclerotic, cardiometabolic and high complexity phenotypes were more often treated with cardiovascular drugs (including ACE inhibitors, diuretics beta-blockers and antiarrhythmics). The cardiometabolic class had also more use of oral hypoglycaemic agents (43.0%), insulin (20.0%) and statins (77.7%). Higher median number of drugs received was observed in the atherosclerotic and cardiometabolic classes (5 [IQR 4–6] in both groups).

Multiple logistic regression models for treatments are reported in Table 2. Compared to the low complexity phenotype, all other groups were associated with higher OAC use, with highest figures in the high complexity (OR [95%CI]: 1.57 [1.35–1.81], *p* < 0.001) and cardiometabolic class (OR [95%CI]: 1.76 [1.49–2.09], *p* < 0.001). Similar results were observed for NOACs, which were more likely used in the high complexity, thromboembolic and ‘CV risk factors’ classes.

**Table 2:** Logistic regression for treatment prescription according to latent classes

Drugs, OR [95%CI]	Low complexity class	CV risk factors class	Atherosclerotic class	Thromboembolic class	Cardiometabolic class	High complexity class
OAC	Ref.	*1.36 [1.24–1.48]*	*1.27 [1.11–1.45]*	*1.48 [1.27–1.72]*	*1.76 [1.49–2.09]*	*1.57 [1.35–1.81]*
NOAC (vs. VKA)	Ref.	*1.21 [1.11–1.31]*	1.03 [0.90*–*1.17]	*1.20 [1.04–1.37]*	1.12 [0.98*–*1.29]	*1.18 [1.03–1.35]*
ACE inhibitors	Ref.	*1.15 [1.07–1.24]*	1.06 [0.94*–*1.19]	1.13 [0.99*–*1.28]	0.99 [0.87*–*1.12]	1.02 [0.90*–*1.15]
Angiotensin receptor blockers	Ref.	*1.13 [1.05–1.22]*	0.96 [0.85*–*1.09]	*0.86 [0.75–1.00]*	1.00 [0.87*–*1.14]	0.97 [0.86*–*1.11]
Diuretics	Ref.	*1.20 [1.11–1.29]*	1.06 [0.94*–*1.20]	0.97 [0.85*–*1.11]	*1.37 [1.20–1.56]*	1.08 [0.95*–*1.22]
Beta-blockers	Ref.	*1.23 [1.15–1.32]*	*1.20 [1.06–1.34]*	*1.16 [1.03–1.31]*	*1.26 [1.11–1.43]*	*1.27 [1.13–1.42]*
Digoxin	Ref.	0.98 [0.87*–*1.11]	*0.77 [0.64–0.93]*	0.88 [0.71*–*1.10]	0.86 [0.70*–*1.04]	1.04 [0.85*–*1.27]
Verapamil/diltiazem	Ref.	1.00 [0.88*–*1.14]	*1.56 [1.25–1.95]*	1.18 [0.93*–*1.49]	*1.84 [1.50–2.26]*	*2.43 [2.02–2.93]*
Class IC antiarrhythmics	Ref.	1.03 [0.90*–*1.17]	0.94 [0.68*–*1.31]	1.00 [0.76*–*1.31]	0.98 [0.71*–*1.36]	0.93 [0.72*–*1.21]
Amiodarone/dronedarone	Ref.	0.97 [0.88*–*1.07]	0.96 [0.83*–*1.12]	0.93 [0.77*–*1.12]	0.88 [0.74*–*1.03]	0.88 [0.75*–*1.04]
Other antiarrhythmics	Ref.	*0.84 [0.72–0.97]*	0.84 [0.68*–*1.03]	0.76 [0.57*–*1.00]	0.85 [0.67*–*1.08]	1.13 [0.90*–*1.41]
Oral hypoglycemic agents	Ref.	*1.42 [1.18–1.71]*	1.24 [0.99*–*1.56]	1.27 [0.92*–*1.76]	*1.41 [1.13–1.77]*	*1.67 [1.29–2.17]*
Insulin	Ref.	0.92 [0.69*–*1.22]	1.09 [0.79*–*1.50]	0.75 [0.44*–*1.26]	*1.43 [1.05–1.94]*	0.99 [0.69*–*1.42]
Proton pump inhibitors	Ref.	*1.20 [1.10–1.30]*	*1.32 [1.16–1.50]*	*1.55 [1.36–1.77]*	*1.66 [1.45–1.90]*	*3.72 [3.30–4.19]*
Statins	Ref.	*4.73 [4.38–5.11]*	*4.35 [3.85–4.92]*	*2.90 [2.55–3.29]*	*8.09 [7.06–9.27]*	*5.35 [4.73–6.06]*

Class IC antiarrhythmics includes propafenone and flecainide. Italic values depict results with *p* < 0.05

*ACE* angiotensin converting enzyme, *OAC* oral anticoagulant, *NOAC* non-vitamin K antagonist oral anticoagulant, *VKA* vitamin K antagonist

Regarding other treatments, all phenotypes showed higher odds of beta-blockers use, compared to the low complexity group. Similar results were observed for verapamil/diltiazem and particularly for the cardiometabolic (OR [95%CI]: 1.84 [1.50–2.26], *p* < 0.001) and high complexity groups (OR [95%CI]: 2.43 [2.02–2.93], *p* < 0.001). Finally, we also found an association between use of non-cardiovascular drugs (oral hypoglycaemic agents, PPI and statins) and more complex phenotypes (Table 2).

### OAC discontinuation

**Fig. 2:**
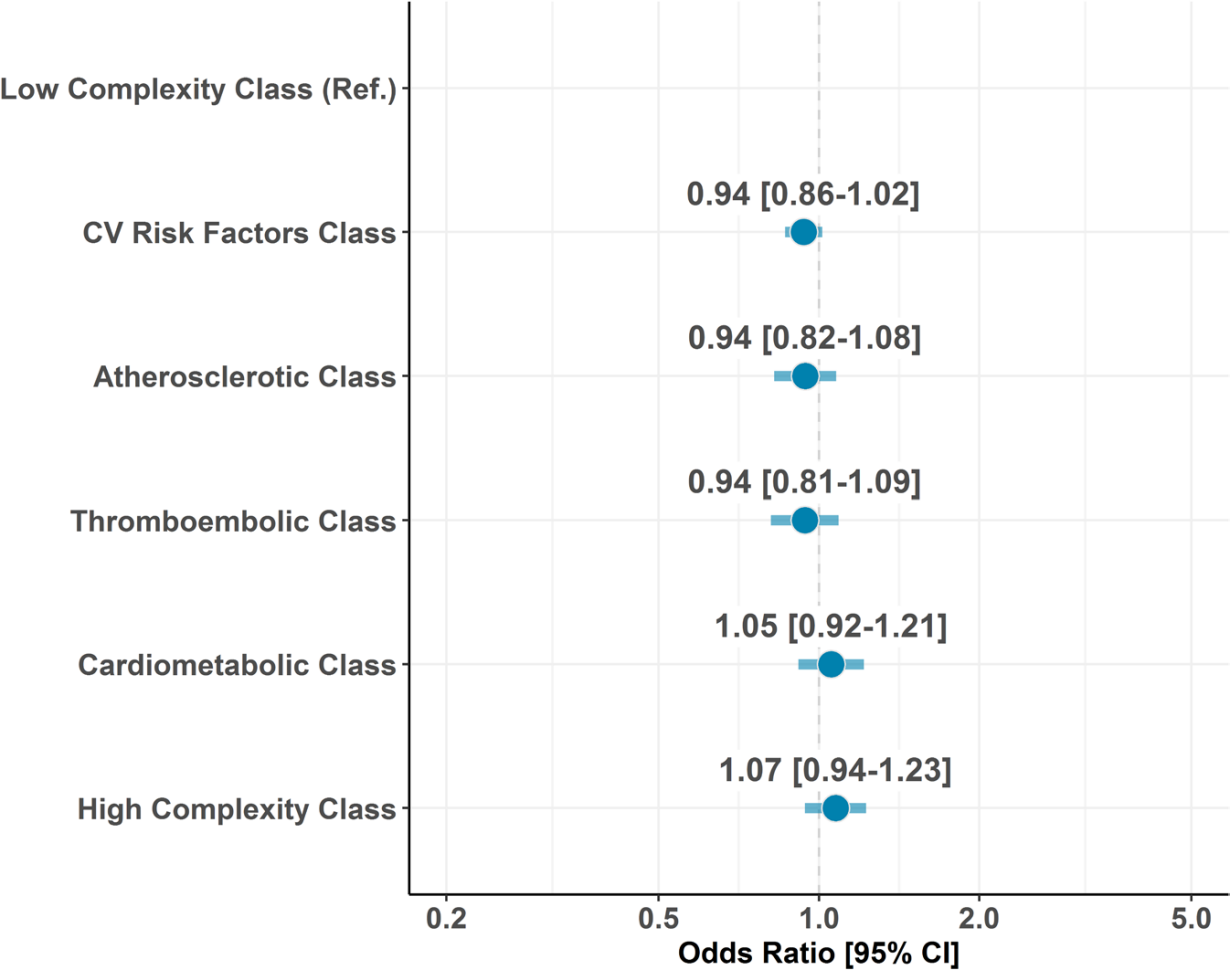
Risk of OAC discontinuation during follow-up according to latent classes

Of the 26,393 patients prescribed OACs at baseline, 19,980 (75.7%) had available follow-up data and were included in the analysis for OAC discontinuation. The proportion of patients who discontinued OAC at 6, 12 and 24 months after enrolment is shown in Additional file 1: Figure S3. Compared to the low complexity phenotype, no statistically significant differences regarding hazard of OAC discontinuation at 2 years were found for the other groups (Fig. 2).

### Risk of adverse outcomes

23,375 patients (71.8%) had follow-up data for the primary composite outcome and were included in the survival analysis. Median follow-up was 3.0 [IQR: 2.2–3.1] years. No statistically significant differences were observed between patients included and excluded regarding age, sex and mean CHA_2_DS_2_-VASc score.

**Fig. 3:**
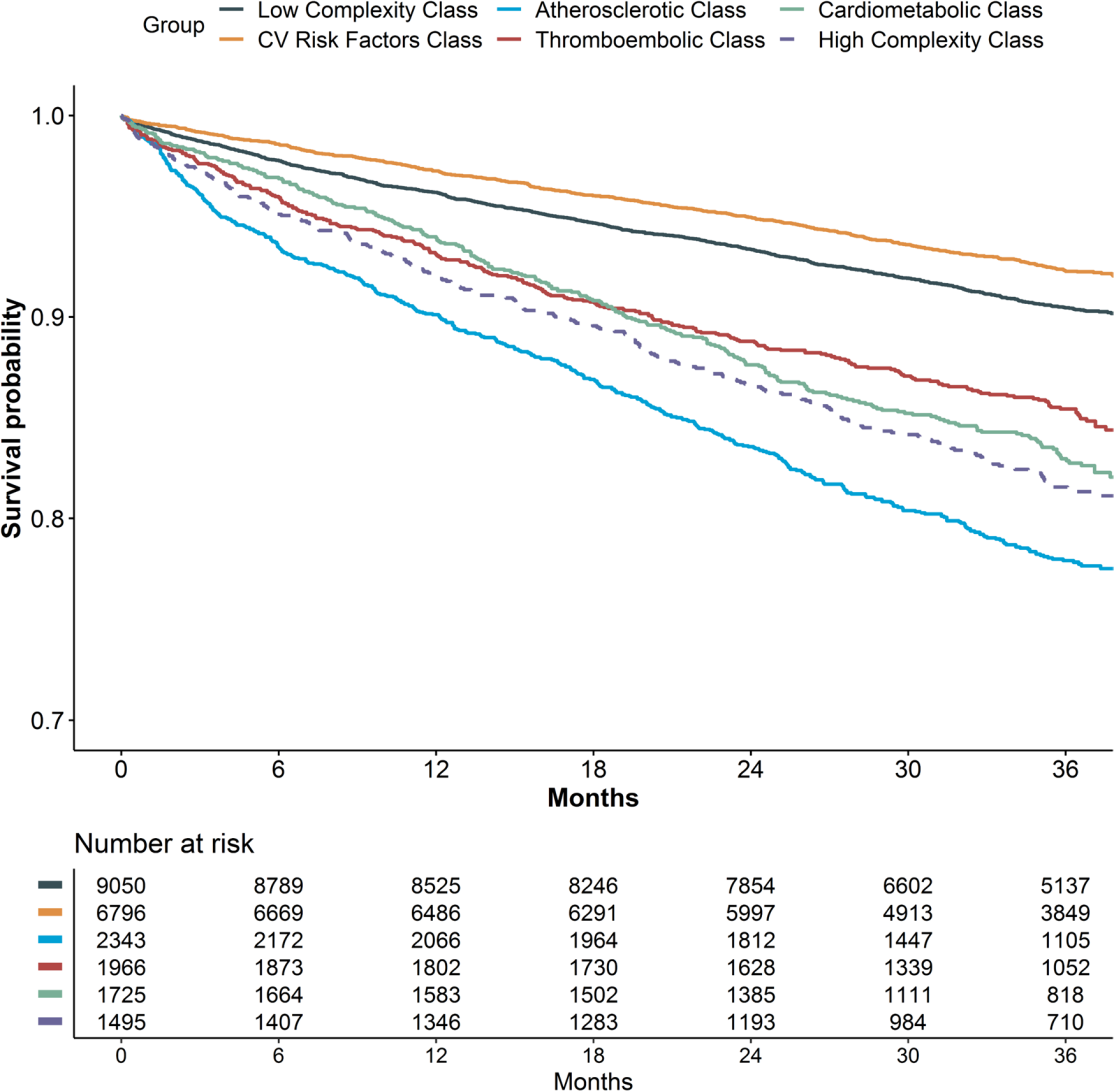
Kaplan–Meier curves for the primary composite outcome of all-cause death and MACE according to latent classes. Log-Rank *p* < 0.001

Kaplan–Meier curves for the primary composite outcome according to latent classes are reported in Fig. 3. The ‘CV risk factor’ and low complexity classes had the highest survival probabilities, while the atherosclerotic and high complexity phenotypes had the highest incidence of the primary composite outcome during follow-up.

Multiple Cox regression models for the primary and the exploratory secondary outcomes are reported in Table 3. Compared to the low complexity phenotype, all other groups were associated with a higher hazard of the primary outcome, except for the ‘CV risk factor’ class (hazard ratio [HR]: 0.87, 95%CI: 0.76–1.00, *p* = 0.042). The greatest association was observed for the cardiometabolic (HR [95%CI]: 1.37 [1.13–1.67], *p* = 0.001) and the high complexity phenotypes (HR [95%CI]: 1.47 [1.24–1.75], *p* < 0.001). Similar findings were observed for all-cause death. The atherosclerotic (aHR [95%CI]: 1.72 [1.24–2.38], *p* = 0.001), cardiometabolic (aHR [95%CI]: 1.77 [1.24–2.51], *p* = 0.001) and high complexity classes (aHR: [95%CI]: 2.19 [1.61–2.97], *p* < 0.001) were also associated with a higher risk of major bleeding, with similar results observed for MACE. Finally, the risk of thromboembolism was higher in the atherosclerotic class (aHR [95%CI]: 1.46 [1.06–2.02], *p* = 0.022) compared to the low-complexity group, while non-statistically significant results were observed for the other groups.

**Table 3:** Multiple cox regressions on the risk of major outcomes according to latent classes

	Low complexity class	CV risk factors class	Atherosclerotic class	Thromboembolic class	Cardiometabolic class	High complexity class
Primary outcome						
Composite of all cause death and MACE						
IR [95%CI]	3.4 [3.2–3.6]	2.6 [2.4–2.9]	8.5 [7.8–9.3]	5.5 [4.9–6.2]	6.4 [5.7–7.2]	6.9 [6.1–7.8]
aHR [95%CI]	Ref.	*0.87 [0.76*–*1.00]*	*1.34 [1.13*–*1.57]*	*1.23 [1.03*–*1.48]*	*1.37 [1.13*–*1.67]*	*1.47 [1.24*–*1.75]*
Secondary outcomes						
All cause death						
IR [95%CI]	2.6 [2.4–2.8]	1.8 [1.6–2.0]	6.5 [5.8–7.2]	4.1 [3.6–4.7]	4.6 [4.0–5.3]	5.6 [4.9–6.4]
aHR [95%CI]	Ref.	*0.83 [0.71–0.97]*	*1.35 [1.13–1.63]*	*1.28 [1.04–1.57]*	*1.33 [1.07–1.66]*	*1.61 [1.33–1.95]*
CV death						
IR [95%CI]	0.9 [0.8–1.0]	0.6 [0.5–0.7]	2.8 [2.4–3.3]	1.1 [0.9–1.5]	1.8 [1.4–2.3]	2.0 [1.6–2.5]
aHR [95%CI]	Ref.	0.81 [0.62–1.06]	*1.37 [1.02–1.85]*	1.19 [0.82–1.71]	1.32 [0.91–1.89]	*1.50 [1.09–2.09]*
MACE						
IR [95%CI]	1.6 [1.5–1.8]	1.4 [1.2–1.6]	5.1 [4.5–5.7]	2.8 [2.3–3.3]	3.6 [3.1–4.2]	3.5 [2.9–4.2]
aHR [95%CI]	Ref.	0.91 [0.75–1.09]	*1.51 [1.20–1.89]*	1.23 [0.96–1.58]	*1.49 [1.14–1.95]*	*1.39 [1.09–1.78]*
Thromboembolism						
IR [95%CI]	0.9 [0.8–1.1]	1.0 [0.9–1.2]	2.0 [1.7–2.4]	2.7 [2.3–3.2]	1.4 [1.0–1.8]	1.8 [1.4–2.3]
aHR [95%CI]	Ref.	0.98 [0.78–1.24]	*1.46 [1.06–2.02]*	1.26 [0.94–1.68]	1.34 [0.91–1.96]	1.35 [0.97–1.88]
Major bleeding						
IR [95%CI]	0.9 [0.8–1.0]	1.0 [0.9–1.2]	1.7 [1.4–2.1]	1.1 [0.8–1.4]	1.9 [1.6–2.4]	2.6 [2.1–3.1]
aHR [95%CI]	Ref.	1.04 [0.82–1.32]	*1.72 [1.24–2.38]*	1.12 [0.77–1.63]	*1.77 [1.24–2.51]*	*2.19 [1.61–2.97]*

Italic text depicts statistically significant results at *p* < 0·05 level

*aHR* adjusted hazard ratio, *CI* confidence Intervals, *IR* incidence rate, *Ref.* reference

## Discussion

In this exploratory analysis from a global and contemporary cohort of AF patients to characterise comorbidity patterns in AF patients, our main findings were (1) comorbidities phenotypes can be found in the general AF populations, each with a specific ‘fingerprint’ and with heterogeneous interplay between cardiovascular and non-cardiovascular comorbidities; (2) comorbidities phenotypes show differences in clinical management, including OAC prescription and choice, rate and rhythm control treatment, and drugs for the treatment of cardiovascular and non-cardiovascular conditions; and (3) patterns of comorbidities were associated with different prognosis.

The epidemiology of comorbidities in patients with AF has been extensively studied, with an emerging growing interest on the topic of ‘clinical complexity’, i.e. the clinical conundrum posed by the co-occurrence of ageing, multimorbidity, polypharmacy and frailty [6, 10, 23], and a recent consensus paper of the European Heart Rhythm Association emphasises the role of frailty and clinical complexity in the natural history of AF patients [24]. Indeed, multimorbidity is a critical driver of clinical complexity [2], but previous research has focused primarily on the cumulative number of diseases [3, 25], rather than on the *patterns* of comorbidities. In this scenario, our analysis represents one of the first attempt to identify groups of AF patients according to their comorbidity patterns, using LCA.

Indeed, in the real-world setting, chronic conditions tend to aggregate and interact, influencing each other. For example, arterial hypertension is known to increase the risk of other cardiovascular diseases, including CAD and CHF [26, 27]; obesity, diabetes and dyslipidaemia are closely intertwined, and each exerts a detrimental effect on the clinical course and progression of the others [26, 28, 29]. More comprehensive and integrated approaches are therefore needed to improve characterisation and management of multimorbidity in AF patients [30, 31], and also to identify potential patterns of comorbidities, that may be managed with specific and targeted interventions, aimed at addressing the underlying complexity, beyond the treatment of the individual diseases.

Taken together, our findings suggest that the overall complexity of AF patients may influence their clinical management. While OAC use increased with phenotype complexity, choice of OAC was heterogeneous, with NOACs being more used for the ‘CV risk factors’, thromboembolic and high complexity classes. Moreover, although the risk of OAC discontinuation was similar between groups, we did not examine drivers of discontinuation which may be different between the phenotype classes [32]. We also observed higher odds of receiving cardiovascular and non-cardiovascular drugs in the ‘CV risk factor’ class, suggesting that more intensive treatment may have contributed to their overall lower risk of major outcomes. Finally, we found higher odds of beta-blocker and verapamil/diltiazem use in more complex classes, with no statistically significant differences for other antiarrhythmic drugs.

While these findings should be interpreted with caution (in view of the potential other clinical indications that may have influenced prescription), our results are consistent with previous studies that showed how older patients and those with more complex clinical profiles were more likely managed with a rate-control approach [33]. These results are important given current evidence supporting effectiveness of early rhythm control strategies, even in AF patients with a high comorbidity burden [34].

We also found that increasingly complex classes were associated with worse prognosis, as shown by the higher risk of the primary composite outcome, and the exploratory secondary outcomes (particularly all-cause mortality). Indeed, risks of thromboembolism and major bleeding were heterogenous across phenotypes: while the atherosclerotic phenotype was associated with increased risk of both outcomes, the highest rates of major bleeding were seen in the high complexity class. This is consistent with the already known detrimental effects of the interaction between modifiable and non-modifiable risk factors on the risk of bleeding [35], the higher risk observed in patients with previous bleeding events and, more generally, in those with increasingly complex comorbidity patterns [6, 35].

While these results should be interpreted with caution, and regarded as hypothesis generating, they suggest that patterns of comorbidities can exert heterogeneous influence on the prognosis of AF, imposing a differential risk of thrombotic and haemorrhagic events, and an overall higher risk of mortality. Indeed, the heterogeneity of underlying complexity in patients with AF may not be optimally characterised by accounting for risk factors in a binary manner (yes/no), given how many diseases commonly occur in combination with each other [36], influencing treatment choices and posing challenges in the management of AF [5, 23, 37]. In this view, our data expand prior observations [10, 36, 38], and provide insights on the identification of clinically meaningful ‘phenotypes’ of AF patients as pivotal for a better risk stratification, and to tailor appropriate management strategies [10, 36, 38]. Individualised and patient-centred care is recommended for patients with cardiovascular diseases, including AF, and particularly in those with several concomitant conditions [39–41]. The ‘Atrial fibrillation Better Care’ (ABC) pathway has been proposed to streamline such an integrated or ‘holistic’ approach to the treatment of AF patients, with a specific focus on the optimisation of treatment of concurrent comorbidities and lifestyle changes [42]. Such approach has been associated with a reduction in the risk of major outcomes [43–46], even amongst AF patients with multimorbidity [47] or those deemed as ‘clinically complex’ [10, 48], and may provide a pragmatic and effective intervention to improve prognosis in patients with complex comorbidity patterns. Further studies will be needed to clarify whether specific and targeted approaches will be able to exert a differential effect on prognosis in patients with different degrees of clinical complexity.

### Strengths and limitations

Our manuscript provides a first application of the LCA approach to analyse comorbidity patterns on a large, contemporary and global real-world cohort of AF patients. These findings inform clinicians on phenotypes of multimorbidity, and implications for management and prognosis. Nonetheless, we acknowledge some limitations. First, the current study is an exploratory post hoc analysis of a prospective observational study; therefore, we may have limited power to find differences between groups. Second, the analysis was based on a set of comorbidities which were available and defined according to the CRF of the GLORIA-AF registry; other diseases, which may be relevant in the natural history of AF, were not included, and we did not analyse the contribution of conditions that were diagnosed after the inclusion. Further studies, appropriately designed, will be needed to analyse the longitudinal trajectories of comorbidities in patients with AF. Moreover, although the median follow-up time in our study was considerable, longer follow-up may be needed to fully capture the trajectories and natural history of patients with AF according to their comorbidity patterns. The GLORIA-AF registry was conducted before the latest international guidelines for the management of AF [31, 39] recommended the implementation of a holistic and integrated approach (such as the ABC pathway) for the management of patients with AF. Therefore, whether more intensive treatment of such comorbidities could have altered our results remains unclear. Although we have adjusted for several covariates, we cannot exclude the contribution of other unaccounted confounders, particularly on the association between treatments and the risk of major outcomes. Finally, our results were not adjusted for multiple comparisons, and as such should be regarded as exploratory and interpreted with caution, particularly regarding secondary outcomes.

## Conclusions

In a large, global and contemporary cohort of AF patients, we identified different patterns of comorbidities, which were heterogeneously associated with clinical management of AF, and with worse prognosis. AF patients with more complex comorbidity profiles may require tailored and integrated approaches to optimise management and improve prognosis. Further studies are required to confirm these results in other settings and cohorts of patients with AF.

### Supplementary Information

**Additional file 1:** **Appendix.** List of GLORIA-AF Investigators. **Table S1.** Diagnostics for Latent Class Models. **Table S2.** Drugs Prescription according to latent classes. **Figure S1.** Workflow of the study. **Figure S2.** Antithrombotic prescribed at baseline according to the latent classes. **Figure S3.** OAC Discontinuation according to the latent classes at 6, 12 and 24 months of follow-up. 

## Data Availability

Data supporting this study by the data contributors Boehringer Ingelheim and were made and are available through Vivli, Inc. Access was provided after a proposal was approved by an independent review committee identified for this purpose and after receipt of a signed data sharing agreement.

## Abbreviations

ACE: Angiotensin converting enzyme
AF: Atrial fibrillation
BIC: Bayesian Information Criteria
BMI: Body mass index
cAIC: Consistent Akaike Information Criteria
CAD: Coronary artery disease
CI: Confidence interval
COPD: Chronic obstructive pulmonary disease
CRF: Case report form
CV: Cardiovascular
GLORIA-AF: Global Registry on Long-Term Antithrombotic Treatment in Patients with Atrial Fibrillation
HF: Heart failure
HR: Hazard ratio
IQR: Interquartile range
LCA: Latent class analysis
MACE: Major adverse cardiovascular events
NOAC: Non-vitamin K antagonist oral anticoagulant
OAC: Oral anticoagulant
OR: Odds ratio
PAD: Peripheral artery disease
SD: Standard deviation
TIA: Transient ischemic attack
VKA: Vitamin K antagonist
